# Stability of Dexamethasone during Hot-Melt Extrusion of Filaments based on Eudragit® RS, Ethyl Cellulose and Polyethylene Oxide

**DOI:** 10.1016/j.ijpx.2024.100263

**Published:** 2024-06-21

**Authors:** Vanessa Domsta, Tessa Boralewski, Martin Ulbricht, Philipp Schick, Julius Krause, Anne Seidlitz

**Affiliations:** a University of Greifswald, Institute of Pharmacy, Biopharmaceutics and Pharmaceutical Technology, Felix-Hausdorff-Str. 3, 17489 Greifswald, Germany; b Heinrich Heine University Düsseldorf, Faculty of Mathematics and Natural Sciences, Institute of Pharmaceutics and Biopharmaceutics, Universitätsstr. 1, 40225 Düsseldorf, Germany

**Keywords:** Hot-melt extrusion, Eudragit® RS, Ethyl cellulose, Polyethylene oxide, Dexamethasone, Degradation

## Abstract

Hot-melt extrusion (HME) potentially coupled with 3D printing is a promising technique for the manufacturing of dosage forms such as drug-eluting implants which might even be individually adapted to patient-specific anatomy. However, these manufacturing methods involve the risk of thermal degradation of incorporated drugs during processing. In this work, the stability of the anti-inflammatory drug dexamethasone (DEX) was studied during HME using the polymers Eudragit® RS, ethyl cellulose and polyethylene oxide. The extrusion process was performed at different temperatures. Furthermore, the influence of accelerated screw speed, the addition of the plasticizers triethyl citrate and polyethylene glycol 6000 or the addition of the antioxidants butylated hydroxytoluene and tocopherol in two concentrations were studied. The DEX recovery was analyzed by a high performance liquid chromatography method suitable for the detection of thermal degradation products. The strongest impact on the drug stability was found for the processing temperature, which was found to reduce the DEX recovery to <20% for certain processing conditions. In addition, differences between tested polymers were observed, whereas the use of additives did not result in remarkable changes in drug stability. In conclusion, suitable extrusion parameters were identified for the processing of DEX with high drug recovery rates for the tested polymers. Moreover, the importance of a suitable analysis method for drug stability during HME that is influenced by several parameters was highlighted.

## Graphical abstract

**Figure:**
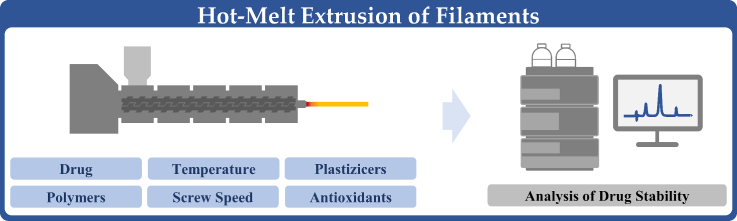

## 1 Introduction

The application of polymeric implants offers the potential of targeted and localized drug delivery while reducing the frequency of medication as well as potential side effects. The manufacturing techniques used to produce implants include compression, solvent casting, hot-melt extrusion (HME), injection molding and more recently 3D printing (Stewart et al., 2018). While the compression method produces implants with a faster drug release profile and the use of large amounts of solvent required for solvent casting raises environmental concerns, the other manufacturing methods of HME, injection molding and 3D printing based on fused deposition modeling (FDM) are thermal processes that involve the risk of drug degradation (Stewart et al., 2018; Domsta and Seidlitz, 2021). Differently shaped implants such as rods, films or structural parts can be produced directly by HME or injection molding, but a fast and highly adaptable process is given by FDM 3D printing, a process that transforms HME into a movement-based construction technology. The 3D printing techniques in general provide enormous potential in the field of individualized medicine. The high degree of freedom in geometrical design offered by these techniques enables the manufacturing of customized dosage forms that meet the patients' needs, for example regarding their size, dose or shape (Bácskay et al., 2022; Dumpa et al., 2021; Elkasabgy et al., 2020). The FDM 3D printing technology is mainly based on a melt extrusion process and uses filaments as feedstock material. Those are molten inside a nozzle and the resulting strands are deposited layerwise in defined pathways on a build plate to form the desired shape by movements in x-, y- and z-direction. HME offers a good opportunity to produce those filaments with a high homogenous drug loading (Shaqour et al., 2020). The majority of current publications deal with FDM 3D printing for oral dosage forms, but drug-eluting implantsare described as well (Domsta and Seidlitz, 2021; Shaqour et al., 2020; Araújo et al., 2019).

Several factors need to be investigated while designing an HME process. Those include for example the chemical and thermal stability, drug-polymer interactions, the rheological properties of the melt or the solid physical state of the extrudates (Censi et al., 2018). All these should be considered in the choice of suitable polymers, drugs and additives for the intended application. Polymers with long-term drug release properties should be applied for the manufacturing of melt extruded or 3D printed implants to enable therapies over periods of weeks to months as well as thermostable drugs for a local treatment such as drugs with antimicrobial or anti-inflammatory activity.

The anti-inflammatory effect of the drug DEX has great potential for the treatment of different diseases as an implant and might be a promising candidate for the manufacturing of 3D printed implants for individualized therapy. This is underlined by the use of DEX in extrusion-based manufacturing processes for implants. For example, DEX is incorporated in the extruded intravitreal implant Ozurdex® approved by the FDA and EMA (U.S. Food and Drug Administration, 2024; European Medicines Agency, n.d.), as well as DEX-containing dosage forms such as implants were already developed by HME or FDM 3D printing in the past (dos Santos et al., 2021; Kelley et al., 2020; Lehner et al., 2019; Farto-Vaamonde et al., 2019; Stein et al., 2018; Li et al., 2018; Costa et al., 2015; Li et al., 2013a; Li et al., 2013b). Moreover, non-thermal 3D printing techniques were already used to produce individually-shaped external ear canal implants or round window niche implant prototypes containing DEX (Matin-Mann et al., 2022; Mau et al., 2022). However, a general processability of DEX by HME or 3D printing including the stability of the drug is not transferable to further developments of hot-melt or 3D printed implants since the used techniques, materials or processing parameters might differ from the intended ones or the stability of the drug has not been analyzed so far. In addition to the thermal exposure caused by the heating elements, mechanical stress from the shear forces could increase potential degradation of excipients during HME. Due to the complexity of the appearing stress on the product, preliminary tests that characterize only the thermal behavior of single or mixed excipients such as thermogravimetric analysis (TGA), would only determine a guidance for temperature conditions related to suspectable thermal degradation, but not an acceptable processing window (Moseson et al., 2020). Consequently, appropriate methods for the quantification of the drug and possible degradation products have to be performed following the HME and 3D printing process to ensure drug stability. The risk of thermal alteration of the drug or further excipients is often present since HME processes are commonly performed at temperatures higher than 150 °C (Shaqour et al., 2020). We have already previously published data on the stability of DEX during the HME process. The findings of that study by our group demonstrated the potential of thermal degradation of DEX during HME and subsequent 3D printing (Krause et al., 2024). In that study compositions mainly based on hydroxypropylmethylcellulose and 1% or 10% (*w/w*) DEX were processed by HME at temperatures of 110–150 °C and subsequent 3D printing of model dosage forms at temperatures of 180–210 °C. Quantification of DEX performed after HME and 3D printing using high performance liquid chromatography (HPLC) indicated degradation of DEX during both processes, especially for HME. The extent of DEX degradation was dependent on the used temperature without a positive effect by the addition of tested antioxidants. Since the processable temperature range for optimal results of the product seems to be much smaller for most polymers in 3D printing than in HME, this initial manufacturing step offers a higher flexibility for process optimization. Consequently, this process is the focus of this study as its impact on the drug stability is of key importance for the final product quality of whether the implants are manufactured customizable by 3D printing or non-customizable directly by HME.

The aim of this study was to continue our previous work (Krause et al., 2024) to examine DEX stability during HME under defined process conditions to investigate the effect of temperature, screw speed and additives while focusing in this study specifically on polymers that are potentially suitable as implant materials. Eudragit® RS (EuRS), ethyl cellulose (EC) and polyethylene oxide (PEO) were identified as suitable polymers for the intended long-term drug release from implants due to the water-insoluble characteristic of EuRS and EC as well as the sustained erosion of high molecular weight PEO as a result of the formed swollen matrix. These polymers were chosen for this work as opposed to the previously tested hydroypropylmethylcellulose (Krause et al., 2024) in order to obtain results for dexamethasone stability in different polymeric implant systems with varying physicochemical properties over a wider range of processing temperatures. These were processed with 10% (*w/w*) DEX to filaments via HME at different temperatures within their processable temperature range and analyzed for DEX recovery as well as the appearance of degradation products. Furthermore, the influence of the screw speed and the addition of triethyl citrate (TEC) or polyethylene glycol 6000 (PEG) as plasticizers as well as tocopherol (Vit E) or butylated hydroxytoluene (BHT) as antioxidants were tested at similar temperatures to determine their influence on the drug stability. A suitable HPLC method was used for the detection of possible degradation products.

## 2 Materials and Methods

### 2.1 Materials

Micronized dexamethasone (DEX; Euro OTC Pharma GmbH, Bönen, Germany) was used as drug and 17-oxo dexamethasone (OXO; Toronto Chemicals Inc., Toronto, Canada) as reference of its main degradation product. The polymers Eudragit® RS 100 (EuRS; chemically: ammonio methacrylate copolymer; Evonik Industries AG, Essen, Germany), ethyl cellulose (EC; ETHOCEL™ Standard 7 Premium; DuPont, Wilmington, USA) and polyethylene oxide (PEO N10 and PEO 303; POLYOX™ WSR N10 with an approximate molecular weight of 100,000 Da, containing 0.8–3.0% silicon dioxide; and POLYOX™ WSR 303 with an approximate molecular weight of 7,000,000 Da, containing 100–1000 ppm BHT and 0.8–3.0% silicon dioxide; The Dow Chemical Company, Midland, USA) were kindly donated by their suppliers. Triethyl citrate (TEC; Merck KGaA, Darmstadt, Germany), polyethylene glycol 6000 (PEG; Carl Roth GmbH + Co. KG, Karlsruhe, Germany), butylated hydroxytoluene (BHT; Sigma-Aldrich Chemie GmbH, Steinheim, Germany) and tocopherol (Vit E; D-alpha, assay: 89%; Caesar & Loretz GmbH, Hilden, Germany) were used as additives. Further chemicals used were formic acid (Carl Roth GmbH + Co. KG, Karlruhe, Germany or VWR International S.A.S., Rosny-sous-Bois, France), methanol (MeOH) and acetonitrile (ACN; both from VWR International S.A.S., Rosny-sous-Bois, France).

### 2.2 Methods

Powder mixtures of different compositions were prepared, processed into filaments via HME and analyzed for drug recovery afterwards. Details on each procedure are described in the following sections.

#### 2.2.1 Preparation of Powder Mixtures

Powder mixtures of different compositions, all containing 10% (*w/w*) of DEX, a polymer base and 0–5% (*w/w*) additives (Table 1), were prepared for HME of filaments suitable for 3D printing application. Since insufficient powder flowability was observed in preliminary tests using Eudragit® RS PO in its powdered form, the granuales of Eudragit® RS 100 were ground (IKA® Tube Mill 100 control, IKA®-Werke GmbH & CO. KG, Staufen, Germany) and sieved for using the particle size class of 250–500 μm. Only polymers with a residual moisture of maximal 1.5% determined by moisture analyzer (*n* = 5; 80 °C; MB35 Moisture Analyzer, Ohaus Corporation, Parsippany, USA) were used for the experiments.

**Table 1:** Compositions of the powder mixtures for hot-melt extrusion (HME) of filaments (proportions in % *w/w*); abbreviations: DEX – dexamethasone, TEC – triethyl citrate, PEG – polyethylene glycol 6000, PEO – polyethylene oxide, Vit E – tocopherol, BHT - butylated hydroxytoluene.

Polymer	Drug	Additive	Mixture Name
Eudragit® RS	10% DEX	–	**EuRS**
		5% TEC	**EuRS_TEC**
		5% PEG	**EuRS_PEG**
Ethyl Cellulose	10% DEX	–	**EC**
		5% TEC	**EC_TEC**
		5% PEG	**EC_PEG**
PEO 303	10% DEX	–	**PEO_303**
PEO (70% PEO 303 + 30% PEO N10)	10% DEX	–	**PEO**
		2.5% Vit E	**PEO_2.5VitE**
		5% Vit E	**PEO_5VitE**
		2.5% BHT	**PEO_2.5BHT**
		5% BHT	**PEO_5BHT**

Powdered components of the compositions were blended using a shaker mixer (Turbula® T2F, Willy A. Bachofen AG, Muttenz, Switzerland) at 49 rpm for 10 min. The addition of liquid Vit E or TEC to the drug-polymer mixture was performed manually in a bowl before mixing in the shaker mixer. Hard aggregates formed by the manual incorporation of the fluid plasticizer TEC were destroyed by grinding the previously frozen (−80 °C at least overnight) powder mixture (IKA® Tube Mill 100 control, IKA®-Werke GmbH & CO. KG, Staufen, Germany) at 25,000 rpm for a few seconds, followed by homogenisation in the shaker mixer. All powder mixtures were prepared a maximum of three days prior to the HME process to minimize any possible interactions between the components in advance.

#### 2.2.2 Hot-Melt Extrusion (HME) Process

**Fig. 1:**
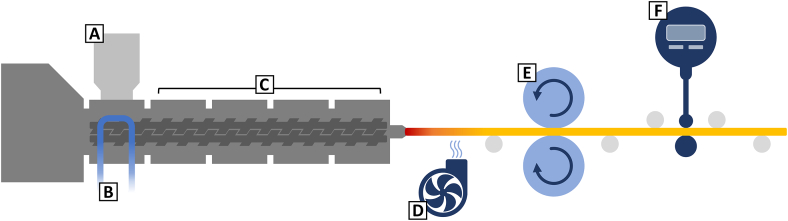
Schematic image of hot-melt extrusion (HME) process. (A) powder feed, (B) water-cooled inlet zone, (C) heating zone 1–4, (D) cooling fan, (E) motorized transport rollers, (F) dial gauge.

The HME process was performed using a co-rotating twin-screw extruder equipped with a flat-tray feeder (ZE 12 with conveying elements (diameter: 12 mm; L/D-ratio 20:1) and ZD 9 FB, Three-Tec GmbH, Seon, Switzerland) and a water-cooled inlet zone (15 °C; CF30 Cryo-compact circulator, JULABO GmbH, Seelbach, Germany). A round-shaped die with a diameter of 2.8 mm for EuRS or EC based compositions and 2.9 mm for PEO based compositions was used for the production of cylindrical filaments in suitable dimensions for 3D printing processes. The extruded filaments were transported from the die by a self-constructed device of rollers and a diameter measurement (Fig. 1). Initially, the hot material is guided by roller bearings and cooled down by fans near the die (D, Fig. 1). The diameter of the extruded filament can be monitored within the process by a digital dial gauge (0.01 mm resolution) equipped with a contact point roller (F, Fig. 1) and be manually adjusted by speed variations of the motorized transport rollers made of silicone (E, Fig. 1).

The same values for feeding rate and preheating of the extruder to constant values before starting the process were used as well as intensive cleaning between each HME process was performed to enable reproducible conditions of manufacturing for each batch. The variation of tested process parameters is listed in Table 2. For the examination of the influence of temperature on DEX stability, HME was performed in a broad range of processable temperatures for each polymer without changing other parameters. Regarding the temperature of the heating zones in front of the die, a range of 110–140 °C was included for EuRS, 150–180 °C for EC and 140–200 °C for PEO in steps of 10 °C. These temperature ranges were chosen after initial tests (data not shown) to determine possible extrusion parameters for each polymer under the specific conditions of the used extruder equipment conditions at the targeted screw speed and material flow. Further influences were examined by HME processes at higher screw speed and with the addition of plasticizers or antioxidants at the same temperature of 120 °C, 160 °C and 170 °C, which already showed good processability for the polymers EuRS, EC and PEO, respectively. Additionally, placebo filaments were prepared from each polymer without DEX.

**Table 2:** Parameters of HME for different compositions containing 10% (*w/w*) dexamethasone.

Mixture Name	Feed Rate (%)	Screw Speed (rpm)	Temperature (°C)		
			Zone 1	Zone 2	Zone 3 + 4
EuRS	2.0	10	70	110	110
	2.0	10	70	110	120
	2.0	10	70	110	130
	2.0	10	70	110	140
	4.0	20	70	110	120
EuRS_TEC	2.0	10	70	110	120
EuRS_PEG	2.0	10	70	110	120
EC	2.0	10	100	140	150
	2.0	10	100	140	160
	2.0	10	100	140	170
	2.0	10	100	150	180
	4.0	20	100	140	160
EC_TEC	2.0	10	100	140	160
EC_PEG	2.0	10	100	140	160
PEO_303	2.0	10	70	140	190
	2.0	10	70	140	200
PEO	2.0	10	100	140	140
	2.0	10	100	140	150
	2.0	10	100	140	160
	2.0	10	100	140	170
	2.0	10	100	140	180
	4.0	20	100	140	170
PEO_2.5VitE	2.0	10	100	140	170
PEO_5VitE	2.0	10	100	140	170
PEO_2.5BHT	2.0	10	100	140	170
PEO_5BHT	2.0	10	100	140	170

#### 2.2.3 Filament Characterization

The visual appearance of extruded filaments was investigated by microscopical imaging using a reflected light microscope (Zeiss Stemi 2000-C with Zeiss CL 1500 ECO, AxioCam ICc 1 and AxioVision software, all Carl Zeiss AG, Oberkochen, Germany). For the quantification of DEX and the detection of potential degradation products via HPLC analysis, filament samples were extracted within the next day after the HME process to exclude potentially storage-associated degradation. Five filament samples of a weight of approximately 100 mg (EuRS, EC) or 50 mg (PEO) were analyzed per batch by taking the samples from different positions of the filament but not the start or end parts related to the filling or emptying phase of the extrusion process. The filament samples based on EuRS, EC or PEO were dissolved in 10 mL of ACN (EuRS), MeOH (EC) or 50 mL of ACN/H_2_O (1 + 1; PEO), respectively, during agitation in a horizontal shaker (KL-2, Edmund Bühler GmbH, Bodelshausen, Germany) at 300 rpm for 24 h at room temperature under the exclusion of light. Frequent vortexing (vortex mixer, VWR International GmbH, Darmstadt, Germany) was performed within the first hours to prevent the formation of a solid sediment. The dissolved samples were diluted with H_2_O (EuRS, EC: first dilution) or ACN/H_2_O (EuRS, EC: second dilution; PEO: one dilution) to suitable concentrations in a solvent matrix of approximately 1 + 1 ACN/H2O, matched to the mobile phase used for HPLC analytics. After each dilution step, the samples were centrifuged (Centrifuge 5702 R, Eppendorf SE, Hamburg, Germany; 4000 rpm, 15 min) to eliminate remaining sediments. This was especially necessary in the case of samples based on EC. The dissolved EC precipitated after the addition of equal volume H_2_O during the first dilution step. Consequently, the supernatant was used for further dilution after centrifugation. Additionally, for each powder mixture five powder samples with masses of 100 mg (EuRS, EC) or 50 mg (PEO) were analyzed accordingly to estimate the status of DEX and degradation products before HME. All analytical samples were stored at −80 °C until analysis.

#### 2.2.4 Thermal stress Tests

Thermal stress tests were performed on pure DEX in the solid state as well as on the other excipients to detect peaks in the HPLC chromatogram related to thermal degradation products and ensure the specificity of the analytical quantification method for DEX. About 500 μg DEX (*n* = 5) were thermally treated in a glass vial in a drying oven (SUT 6120, Heraeus Instruments GmbH, Hanau, Germany) for 1 h at temperatures of 100–200 °C in 10 °C intervals as well as for 6 h at 200 °C. After cooling the drug was dissolved in ACN/H_2_O and analyzed by HPLC. Additionally, further excipients were treated similarly at 200 °C for 1 h and 6 h.

#### 2.2.5 HPLC Analysis

The determination of DEX and possible degradation products was performed from dissolved samples using a Shimadzu Nexera LC40 HPLC system (Shimadzu Corporation, Kyoto, Japan) consisting of a LC-40B XR solvent delivery module, a DGU-403 degassing unit, a SIL-40C XR autosampler, a CTO-40S column oven and a SPD-M40 photodiode array detector. The HPLC system was operated with the software LabSolutions (version 5.99, Shimadzu Corporation, Kyoto, Japan). Previously described methods (Mau et al., 2022; Krause et al., 2024; Ulbricht et al., 2022) were adapted especially regarding the complete elution of lipophilic excipients and the adapted analytical method was validated with respect to linearity, precision, accuracy, specificity, freeze-thaw and rack stability following the ICH guideline “Validation of Analytical procedures Q2 (R1)”. A Phenomenex Kinetix® C8 column (150 × 2.1 mm, 2.6 μm, equipped with an according precolumn, Phenomenex Inc., Torrance, USA) was used for the separation of DEX, further excipients and degradation products. The column was temperated at 40 °C. Samples were injected in a volume of 5 μL and detected at a wavelength of 240 nm. A gradient method (Table 3) with water containing 0.1% formic acid (A) and ACN (B) as mobile phase was used at a flow of 0.5 mL/min. The retention time of DEX was 12.7 min. The quantification range of DEX was 4–80 μg/mL. Concentrations of 0.4–40 μg/mL OXO were analyzed to determine the retention time, signal-to-concentration linearity and relative response factor of this degradation product. Additionally, all excipients were analyzed in unstressed conditions and after thermal treatment at 200 °C for 1 h and 6 h.

**Table 3:** Composition of mobile phase during gradient method (A: H_2_O with 0.1% formic acid; B: ACN).

Time (min)	Mobile Phase A	Mobile Phase B
0	80	20
13.5	80	20
22.5	10	90
25.5	10	90
27.5	80	20
32.0	80	20

The performed HPLC analysis was suitable for the quantification of DEX at a retention time of 12.7 min. Neither degradation products of DEX nor other excipients under thermal stress or unstressed conditions eluted near the DEX peak. The degradation product OXO was identified by the known retention time of the OXO reference at 17.1 min. Additionally, a relative response factor of 1.12 could be estimated for OXO as the ratio of the slope of OXO and DEX, whereas the slopes were determined by the linear regression equation for peak area vs. concentration, respectively. Consequently, the percentage peak areas (%^A^) of the components DEX, OXO and unknown degradation products without the inclusion of additives were calculated to represent their fractions.

## 3 Results

### 3.1 Visual Appearance

**Fig. 2:**
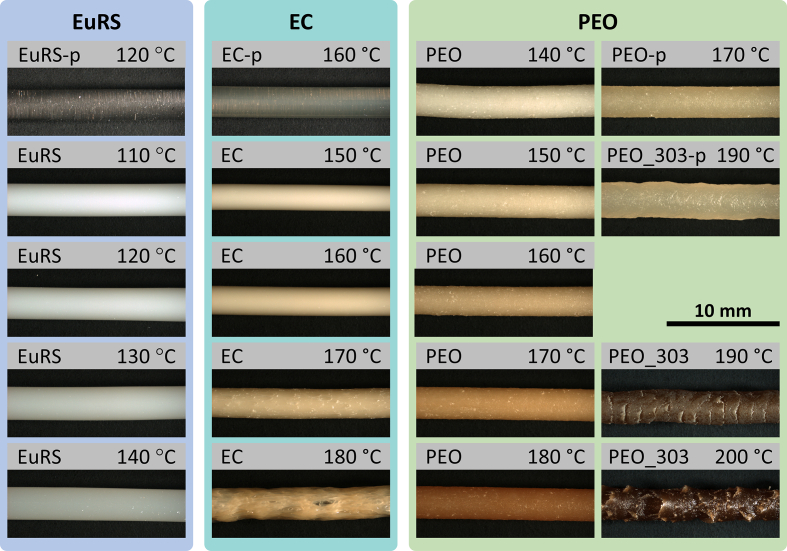
Microscopic images of filaments based on EuRS, EC and PEO containing 10% (*w/w*) dexamethasone or no drug (p). HME of these filaments was performed at 10 rpm (screw speed) and different processing temperatures (heating zone 3 + 4).

Representative microscopic images are illustrated in Fig. 2 for placebo filaments and filaments containing 10% (*w*/w) DEX without any additives. The color of filaments that were extruded at higher screw speeds of 20 rpm seemed (data not shown) to be minimally lighter compared to filaments of the same composition processed at the same temperature but at 10 rpm screw speed. An essential optical difference between the filaments with and without additives was not detected for the same processing temperatures and screw speed. All DEX-loaded filaments had an opaque appearance. However, the placebo filaments showed slight to complete transparency. The placebo EuRS filament was colorless and the filaments containing DEX were colored white without substantial difference regarding different processing temperatures. The filaments based on EC showed color changes starting with a yellowish-white color at 150 °C getting darker with increasing extrusion temperature accompanied by broken surface structures. Similar color changes without alterations in surface structure were observed for the EC placebo filaments at temperatures from 150 °C to 170 °C. The most intensive change in color was observed for PEO based filaments containing DEX. Those filaments had a yellowish-white appearance at extrusion temperatures of 140 °C, but darkened up to a brownish-black color at 200 °C with disrupted surface.

### 3.2 Thermal Stability of Dexamethasone

**Fig. 3:**
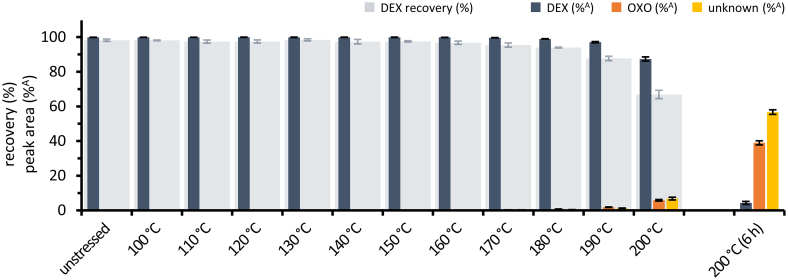
Recovery of dexamethasone unstressed and after thermal treatment. Recovery of dexamethasone in % based on the theoretical content (DEX recovery), and percentage peak area of DEX (%^A^), OXO (%^A^) and unknown degradation products (%^A^), mean ± SD, *n* = 5.

The thermal degradation of pure DEX in the solid state is illustrated in Fig. 3 as percentage recovery of the theoretical content, starting at 98.2 ± 0.7% under unstressed conditions. Additionally, the percentage peak areas of DEX, the main degradation product OXO and other unknown degradation products are represented. The peak at a retention time of 17.1 min, identified as degradation product OXO, increased with samples treated at higher temperatures from 0.2 ± 0.1%^A^ at 170 °C to 5.8 ± 0.5%^A^ at 200 °C for 1 h. Peaks at retention times of 16.2 min, 17.1 min and 17.5 min were clearly visible beside small deviations from the baseline in samples treated for 6 h at 200 °C. In accordance to the decrease of DEX detected by HPLC, color changes of the DEX powder from white over yellow to brown were visually observed starting at 170 °C and intensified with increasing temperature. The brown-colored product after heat treatment at 200 °C for 6 h was not completely soluble in the dissolution media. Consequently, the HPLC data represents only the remaining soluble components, where DEX recovery was <1% of the theoretical content.

### 3.3 Influence of Hot-Melt Extrusion

The influence of the HME process on the stability of DEX was determined in comparison to the initial state in the powder mixtures. The mean recovery of DEX in the powder mixtures of each batch ranged from 89.5% to 105.7% and no degradation products were detected.

Compared to the results of the percentage DEX area, higher variations in DEX recovery values were observed in relation to the theoretical drug content of 10% (*w*/w). This is influenced by variations in the distribution of the drug in the powder mixtures and filaments, as well as by slight variations related to the sample preparation. Consequently, the percentage peak areas of DEX, OXO and unknown degradation products were primarily used for an interpretation of the proportion between DEX and the degradation products independently of those variations. Peak areas related to blank impurities or added excipients were excluded from these calculations.

#### 3.3.1 Influence of Temperature and Polymer

**Fig. 4:**
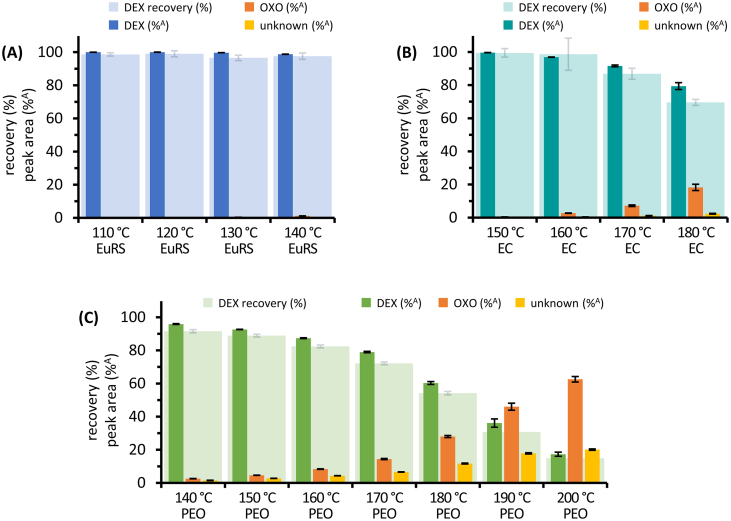
Recovery of dexamethasone in filaments based on EuRS, EC or PEO manufactured at different extrusion temperatures. Recovery of dexamethasone in % based on the theoretical content (DEX recovery), and percentage peak area of DEX (%^A^), OXO (%^A^) and unknown degradation products (%^A^), mean ± SD, *n* = 5.

The recovery of DEX as well as the quantitative relation of drug and degradation product, represented as percentage peak area, are illustrated in Fig. 4 for filament samples based on EuRS, EC and PEO at different extrusion temperatures.

The filaments based on EuRS which were extruded in a temperature range of 110–140 °C showed only a minimal decrease of DEX to 98.8 ± 0.1%^A^ at 140 °C accompanied by minimal amounts of detected OXO (Fig. 4A). A continuous decrease of DEX combined with an increase in degradation products is visible for filaments based on EC or PEO, processed at temperature ranges of 150–180 °C or 140–200 °C, respectively (Fig. 4B,C). A tremendous reduction of DEX to only 17.3 ± 1.3%^A^ was detected at the highest temperature of 200 °C in PEO samples. Furthermore, differences in the degree of degradation were identified by comparing the results for different polymers at the same temperatures in the range of 140–180 °C. Higher amounts of degradation products were detected for PEO samples compared to EuRS or EC samples processed at the same temperatures.

#### 3.3.2 Influence of Screw speed

**Fig. 5:**
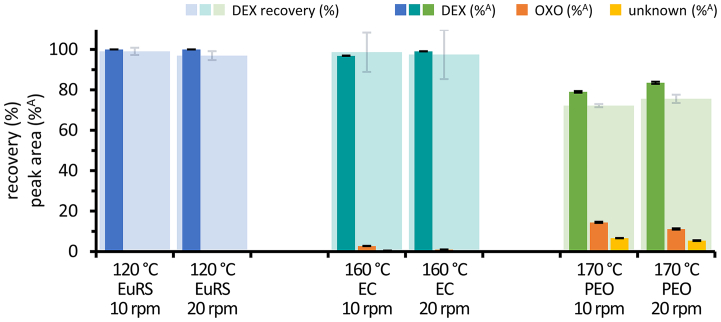
Recovery of dexamethasone in filaments based on EuRS, EC or PEO manufactured at screw speeds of 10 rpm and 20 rpm. Recovery of dexamethasone in % based on the theoretical content (DEX recovery), and percentage peak area of DEX (%^A^), OXO (%^A^) and unknown degradation products (%^A^), mean ± SD, *n* = 5.

Comparing the DEX recovery values of filaments of EuRS and EC processed at screw speeds of 10 rpm and 20 rpm at the same temperatures, negligible degradation was observed for both (Fig. 5). Regarding the results of PEO based samples, the degradation of DEX seemed to be slightly decreased with higher screw speed since the non-degraded DEX fraction was 83.5 ± 0.6%^A^ using 20 rpm and 79.0 ± 0.4%^A^ at 10 rpm.

#### 3.3.3 Influence of Plasticizers and Antioxidants

**Fig. 6:**
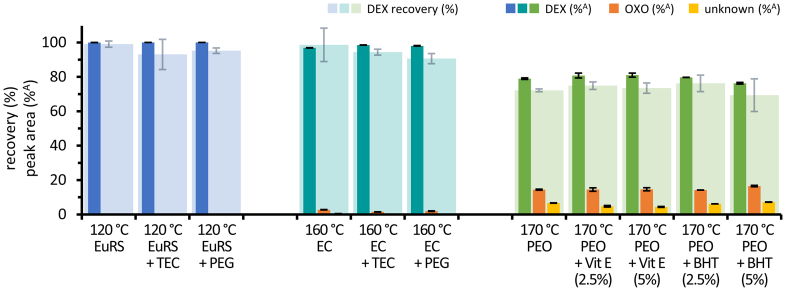
Recovery of dexamethasone in filaments based on EuRS, EC or PEO with different additives. Recovery of dexamethasone in % based on the theoretical content (DEX recovery), and percentage peak area of DEX (%^A^), OXO (%^A^) and unknown degradation products (%^A^), mean ± SD, *n* = 5.

Neither the addition of plasticizers TEC or PEG to EuRS or EC filaments nor the addition of the antioxidants BHT and Vit E in concentrations of 2.5% or 5% to PEO filaments, resulted in intensive differences compared to filaments processed under the same conditions without additives (Fig. 6). Apparent differences in DEX recovery were not confirmed by comparing the results of percentage peak areas. At the tested temperatures, the mean values of DEX fraction ranged between 99.97 and 100.00%^A^, 96.9–98.5%^A^ and 76.3–81.0%^A^ for EuRS, EC and PEO based filaments with and without additives, respectively.

## 4 Discussion

Melt extrusion based manufacturing processes, including HME, injection molding and 3D printing are well suitable techniques for the manufacturing of polymer-based drug eluting implants. Especially, FDM 3D printing coupled with HME process has high potential for the manufacturing of customized drug-eluting implants due to the great freedom of geometrical shapes fitted to the anatomical structures of the individual patients that can be achieved by this procedure. A prerequisite for the development and optimization of a suitable FDM 3D printing process is the production of high quality filaments via HME. In this study, the manufacturing of filaments was successful for the tested polymers EuRS, EC and PEO loaded with 10% *(w/w)* dexamethasone in their processable temperature ranges by HME. A suitable powder flowability, achieved by adjusting the particle size for EuRS, enabled less fluctuation in material transport during HME process. Furthermore, degassing during HME might improve the homogeneity of the process and a reduced moisture content might facilitate the production of extrudates with a consistent and uniform appearance (Alshahrani et al., 2015). Unfortunately, degassing during HME was not feasible due to the absence of vents on the extruder equipment in use. Consequently, the use of polymers with limited residual moisture was able to minimize vapor formation within the extruder barrel and the final filaments were produced homogeneously.

HME, injection molding as well as 3D printing are manufacturing techniques, which imply a higher risk of degradation of drugs or further excipients due to their thermal processing conditions. The degradation of different drugs during HME or FDM 3D printing has already been described for different polymers and processing temperatures (Krause et al., 2024; Hoffmann et al., 2023; Kempin et al., 2018; Hengsawas Surasarang et al., 2017; Goyanes et al., 2016; Goyanes et al., 2015; Thumma et al., 2008a; Repka et al., 1999). Therefore, it is essential to ensure an acceptable level of stability of all excipients, particularly the drug, for the intended compositions and processing parameters during the manufacturing process. In cases in which instability due to oxidation is observed, the first potential countermeasure that comes to mind is the addition of antioxidants to protect the drug or other excipients. The focus of this study was the determination of the drug content in the different polymeric formulations under different processing conditions, as its intact state is essential for the therapeutic effect. However, the analysis of potential degradation products related to the polymer or further excipients will be required in the future steps of implant development, particularly to ensure that biocompatibility and drug release properties are not negatively affected.

The HPLC-based method used in this study was suitable to analyze the drug stability by quantification of DEX and the detection of degradation products. The thermal stress test on pure DEX in the solid state indicated degradation of the drug beginning at 170 °C with the detection of the main degradation product OXO after thermal treatment of 1 h. Thermally-induced oxidative degradation of DEX to OXO seems to be the predominant mechanism but further unspecified degradation products occurred at higher temperatures and longer durations of thermal treatment. These findings differ from an exclusive detection of OXO as degradation product after oxidative stressing of DEX in solution as described by Chen et al. (Chen et al., 2008) and may underline the need for differential interpretation of thermal drug degradation in solid state compared to solutions. Since the further degradation products related to the peaks appeared after intensive thermal treatment were not identified in this study, their chemical structure and the mechanism of degradation starting from DEX with OXO as a possible intermediate is not clear. According to the literature, onset temperatures for mass loss of DEX are reported between 210 °C and 256.6 °C from TGA studies (Santos et al., 2021; Oliveira et al., 2018; Jain and Datta, 2015). These temperatures are higher than the temperatures at which degradation products were identified in this study during the thermal stress test or HME. Although TGA is frequently used for the determination of acceptable processing temperatures for HME, this method is limited assessing the processability of a drug to certain HME conditions as discussed by Moseson et al. (Moseson et al., 2020). Depending on the performed method parameters, such as the heating rate, or detection method, they identified varying degradation onset points in wide ranges with up to 170 °C difference using this method. The thermal treatments of a TGA experimental setup, usually performed under an inert atmosphere may not be comparable to those of an extrusion process, where contact of the melt with oxygen is not completely avoidable. The thermal treatment of the drug in solid state under atmospheric conditions and subsequent HPLC analysis may be an alternative analytical method for the estimation of drug stability during HME. Nevertheless, the validity of this method is also limited since the aspects of exact HME duration, mechanical stress and interactions of drug with polymers or additives are not adequately addressed. However, this type of thermal stress test on DEX and all excipients enabled the confirmation of the specificity of the used HPLC analysis for the performed studies since DEX peak was separated from all further peaks related to the excipients or their thermal degradation products.

Since the relative response factor of OXO was close to 1.0, the analysis of percentage peak areas should be equivalent to the mass fractions of OXO and DEX without the need of corrective calculations, which are usually applied when the relative response factor is outside the range of 0.8–1.2 (EDQM Council of Europe, 2024; Épshtein, 2019; Bhattacharyya et al., 2005). The percentage peak area of unknown peaks was similarly calculated although the relative response factor was not known for these. This method enabled the comparison of the fractions of undegraded DEX and its degradation products in each batch independently of variations in tested samples due to possible inhomogeneities in drug distribution or variations in sample preparation, as indicated by higher variations of the mean and standard deviation of DEX recovery results compared to the theoretical drug content of 10% *(w/w)*. However, the interpretation of the results exclusively based on the percentage peak area seems to slightly underestimate the DEX degradation as values of DEX recovery related to the theoretical drug load were often lower than the results of percentage peak area. A reasonable explanation for these finding might be an underestimation of unknown degradation products due to the unknown response factor or degradation products with insoluble or undetectable properties. Nevertheless, the influence of the different processing parameters could be assessed with this evaluation method, but it should probably not be used exclusively to confirm the stability of the drug.

Regarding the results of this study, the processing temperature seems to be the most important parameter affecting the stability of DEX during HME. Increased degradation of DEX was observed with increasing temperatures up to a DEX recovery below 80%^A^ for EC based filaments at 180 °C and below 20%^A^ for PEO based filaments at 200 °C. At lower temperatures between 110 °C and 140 °C as used for EuRS based filaments DEX was recovered above 98%^A^. Processing temperatures for HME or FDM 3D printing of DEX embedded in different polymers are described in the literature in a wide range of 40–220 °C (dos Santos et al., 2021; Kelley et al., 2020; Lehner et al., 2019; Farto-Vaamonde et al., 2019; Stein et al., 2018; Li et al., 2018; Costa et al., 2015; Li et al., 2013a; Li et al., 2013b). Since the analysis of drug stability or detection of degradation product was not addressed explicitly by these studies, the drug content was not examined or spectrophotometric analysis was performed, which may not differentiate between DEX and degradation products with similar molecular structure and absorbance properties. Consequently, the stability of DEX during the described processes is not always known. However, Lehner et al. (Lehner et al., 2019), Santos et al. (dos Santos et al., 2021) and Stein et al. (Stein et al., 2018) processed DEX without reporting any drug degradation after HPLC analysis of the extrudates or printed objects. The stability of DEX in these products is probably caused by the low processing temperatures of 50–52 °C, 90–105 °C or 40–56 °C, respectively, used in these processes, which is consistent with the results of this study. The considerably higher temperatures used in this study resulted from experiments regarding the processability of the chosen combinations of the drug and excipients with the available extruder equipment and screw configuration. Potentially, other setups might allow for different processing temperatures, screw speeds and residence times of the materials in the extruder and might therefore also lead to different outcomes regarding stability.

A general dependency of drug stability on the processing temperature during HME is supported by the findings of Thumma et al. (Thumma et al., 2008a) and Repka et al. (Repka et al., 1999), where drug recovery increased with decreased processing temperatures of the same composition. Moreover, the results of the presented study highlight the influence of the used polymer in addition to the processing temperature. The DEX recovery of PEO based filaments processed at 140 °C was lower than that of EuRS based filaments processed at the same temperature as well as the DEX recovery was reduced in PEO based filaments accompanied by higher portions of unknown degradation products compared to EC based filaments processed in the temperature range of 150–180 °C. The oxidative degradation of DEX in PEO based filaments may be enhanced by the thermal-oxidative degradation of the PEO itself (Chen et al., 2016) and the polymeric degradation products could potentially cause further degradation of DEX and OXO, resulting in higher levels of unknown degradation products. The identification of further detected degradation products by appropriate analytical methods, e.g. mass spectrometry, in the future would be useful to understand the details of the degradation mechanism in this case. In addition to drug-polymer interactions, different conditions in the polymer melt might be responsible for the observed differences. Different melt viscosities are probably apparent when processing different polymers at the same temperature. This would result in different shear stresses forcing the composition and consequently affecting the degree of drug degradation.

These results highlight the influence of the polymer on the drug stability during HME. Thus, no specific temperature range without drug degradation could be determined. The different physicochemical properties of the tested polymers influence the HME process as well as the properties of the final dosage form. According to the manufacturer information EuRS has a glass transition temperature of 58 °C (*Evonik Industries AG, EUDRAGIT® Application Guidelines, 1.3. GLASS TRANSITION TEMPERATURE (TG*), 2024), EC of 129–133 °C (*The Dow Chemical Company, Ethocel - Ethylcellulose Polymers*, 2005) and PEO has a crystalline melting point at 62–67 °C remaining a high degree of crystalline character for some types far above this temperature (*The Dow Chemical Company, Polyox - Water-Soluble Resins*, 2003). The different softening ranges as well as the melt viscosity and chemical reactivity of the polymers have an impact on the HME process and thus on the stability of the incorporated drug that is forced to withstand different thermal, mechanical and chemical stresses. Furthermore, the properties of the manufactured implant in terms of solubility, lipophilicity, swelling behavior and mechanical properties are determined by the choice of polymer, which already limits the applicable processing temperature. Consequently, the implant will have different suitability for different application sites depending on the polymer used. The insoluble polymers EuRS and EC would remain in place while the water soluble PEO dissolves over time. This could be advantageous if the implant is not intended to be permanent and resections would be avoided by dissolution of the implant, but for implants with mechanical stabilization tasks EuRS and EC may be suitable. In addition, the studies by Kempin et al. (Kempin et al., 2017) have already demonstrated long-term drug release over more than two months from 3D printed implants based on EuRS and EC. The exact release characteristics of the formulations described in the current study will probably differ since the used drug, drug content and EC type were different. However, the potential for long-term use has been demonstrated, probably not being feasible to this extent by the water soluble PEO based implants. Since the polymers would be selected mainly for the intended purpose, it is essential to determine the stability of the drug in the specific formulation of drug, polymer and potential additives by using an appropriate method.

Regarding the visual appearance of the processed filaments increased DEX degradation is related to discolorations of the filament over yellowish to dark brown. Consequently, the visual inspection of filaments is a simple indicator to detect possible instabilities, similarly detected by Kempin et al. (Kempin et al., 2018) in the darkening of pantoprazole sodium containing filaments processed above the degradation temperature. Nevertheless, it should be stated that it is not possible to clearly attribute the discoloration to the degradation of drug, polymer or additives themselves. According to experiments on HPMC of Prasad et al. (Prasad et al., 2019) and own findings on EC, a darkening of the pure polymer is visible with increased temperatures or shear stress. In contrast, the placebo filaments based on PEO had similar color at temperatures of 130–200 °C, but the dark color is intensified with increasing temperature in the case of DEX-loaded filaments. In this instance, the discoloration might be related exclusively to the degradation of DEX.

Moreover, increased drug degradation during HME is reported with extended heating duration as well as higher screw speeds, which is related to stronger mechanical shear forces (Hengsawas Surasarang et al., 2017; Thumma et al., 2008a; Huang et al., 2016). On the other hand, a faster feed rate decreased degradation due to reduced residence times (Huang et al., 2016). No correlation between screw speed and the stability of DEX was found in the performed extrusion experiments with doubled feed rate and screw speed for EuRS and EC based filaments as the DEX recovery was high and only differed slightly. The degradation of DEX in PEO based filaments, processed at 170 °C at 10 rpm seemed to be reduced with the higher screw speed of 20 rpm that is associated with a reduced residence time of the composition in the heated extruder. Since the increase in the DEX fraction was only a small percentage, further tests at even higher screw speeds should be performed to confirm this protective trend and to estimate limits regarding the HME processability for uniform filaments at highly accelerated screw speeds.

The addition of plasticizers is commonly used to improve the processing conditions during HME. Additives with low molecular weight or the drug itself can act as plasticizers by enlarging the free volume in the polymer chains (Douroumis, 2012; Crowley et al., 2007). Citrate esters, fatty acid esters, sebacate esters, phthalate esters or glycol derivates are examples for commonly used plasticizers in pharmaceutical dosage forms (Douroumis, 2012; Crowley et al., 2007). These decrease the glass transition temperature and thus enable lower processing temperatures to prevent degradation processes, improve the flow of the melt or reduce the brittleness of the final product (Douroumis, 2012; Crowley et al., 2007). Kempin et al. (Kempin et al., 2018) used this strategy in HME and 3D printing of the thermo-labile drug pantoprazole sodium to enable lower extrusion temperatures and Thumma et al. (Thumma et al., 2008a) detected lower degradation of Δ^9^-tetrahydrocannabinol-hemiglutarate by the addition of different plasticizers to the formulation processed at the same temperature. However, depending on the type and concentration, plasticizers may also affect the mechanical properties of the product, the drug release properties or the stability of the drug or dosage form during storage (Thumma et al., 2008a; Schilling et al., 2010; Zhu et al., 2006). Thus, several aspects need to be considered in the selection and use of plasticizers. The tested plasticizers in this study, TEC and PEG, were added to EuRS and EC based compositions. No perceptible decrease of DEX was observed compared to the plasticizer-free compositions extruded at the same temperature. A potential positive influence of these plasticizers on DEX stability could not be assessed in this setting due to high recovery values above 96%^A^ DEX in the extrudates with and without plasticizer addition. Nevertheless, these plasticizers seem to be applicable in HME with DEX without adversely affecting drug stability at the tested temperatures.

The incorporation of plasticizers may offer advantages by lowering the HME processing temperature and thereby increasing the distance to the degradation temperature. A reduction of the processing temperature might also stabilize DEX in PEO based filaments. The unplasticized PEO composition would probably be processable at temperatures below 140 °C since the maximum torque was not reached during the HME process at this temperature. However, the addition of plasticizers or increase of the PEO N10 content might allow even lower processing temperatures.

Additionally, plasticizing would be even more relevant for subsequent 3D printing, which is usually performed at higher temperatures compared to HME. Furthermore, the reduced melt viscosity may improve the material flow during 3D printing and thus enable more accurate deposition of material (Domsta et al., 2023). Nevertheless, highly flexible filaments as a result of higher plasticizer amounts would cause difficulties regarding their feeding behavior in 3D printing and might be not processable using this technique (Shaqour et al., 2020; Hassim et al., 2021; Nasereddin et al., 2018). Moreover, the used plasticizers have an impact on the mechanical properties of the final product. Considering the variety of application fields for a drug-eluting implant, such as cardiovascular, gynecological, orthopedic, dental, ophthalmic or cochlear (Quarterman et al., 2021), these modifications caused by plasticizers may or may not be desirable depending on the specific application.

Oxidation seems to be the predominant degradation mechanism of DEX during HME. The evaluation of the oxidation sensitivity of a drug or composition in the solid state is not easily feasible due to the current lack of a best practice protocol for studying oxidative degradation in this state (Waterman et al., 2002). If oxidative degradation of the processed drug is analyzed or suspected, the addition of antioxidants may reduce the rate of degradation. Examples of preventive or chain-breaking antioxidants commonly used in pharmaceutical dosage forms are ascorbic acid, ethylenediaminetetraacetic acid (EDTA), citric acid, sulfites and hindered phenols, such as butylated hydroxyanisole, BHT or Vit E, which protect the drug by autooxidation, chelation or inhibition of free radical chain reactions (Crowley et al., 2007; Hovorka and Schöneich, 2001).

The application of antioxidants in HME or 3D printing described in the literature has addressed different intentions. The incorporation of lignin into polylactic acid or polybutylene succinate as active ingredient by Domínguez-Robles et al. (Domínguez-Robles et al., 2019) and Abdullah et al. (Abdullah et al., 2022) enabled 3D printing of dosage forms with antioxidative capability. Crowley et al. (Crowley et al., 2002) and Repka et al. (Repka and McGinity, 2000) tested different antioxidants to reduce oxidative degradation of the polymer PEO, whereas Thumma et al. (Thumma et al., 2008b) and our previously performed study (Krause et al., 2024) intended the protection of the active ingredient, a prodrug of Δ^9^-tetrahydrocannabinol or DEX, respectively. Most antioxidants were tested in concentrations between 0.05% and 5%, but D-α-tocopheryl polyethylene glycol 1000 succinate was added up to 30%. Reduced degradation of drug or polymer was demonstrated in these studies for the addition of D-α-tocopheryl polyethylene glycol 1000 succinate, Vit E succinate, Vit E, BHT, propyl gallate and EDTA in the tested concentrations. However, ascorbic acid, BHA and Vit E acetate did not stabilize the molecular weight of the polymer PEO, but lower to similar concentrations of ascorbic acid and BHA reduced the degradation of the analyzed prodrug Δ^9^-tetrahydrocannabinol-hemiglutarate. These inconsistent results demonstrate that the protective potential of antioxidants depends not only on the type and concentration of the antioxidant, but is affected by the overall system. Furthermore, since these additives would also affect the melt viscosity, especially at higher concentrations, the antioxidant effect cannot be considered in isolation.

In the presented studies on the stability of DEX in PEO during HME at 170 °C similar levels of degradation were observed by the addition of 2.5% or 5% of BHT or Vit E in comparison to compositions without antioxidants. Similar DEX degradation without a positive influence by the addition of antioxidants BHT, Vit E and ascorbic acid in the concentration of 0.2–2.5% was described by Krause et al. (Krause et al., 2024) for HME and 3D printing based on the polymer hydroxypropylmethylcellulose. Since the dependence of the antioxidant concentration on the extent of degradative prevention has been described in the literature, different results may be obtained with higher amounts of antioxidants but would be unlikely considering the successful antioxidative capacity of BHT and Vit E described for concentrations multiple times lower that reduced polymer degradation or increased storage stability of hot-melt extruded oral or transmucosal dosage forms (Crowley et al., 2002; Thumma et al., 2008b). An aspect that possibly adversely affected the antioxidative stabilization of DEX in tested conditions, might be the reported thermal instability of multiple antioxidants associated with a partial inactivation (Santos et al., 2012; Reda, 2011; Sabliov et al., 2009; Sanhueza et al., 2000). The onset of inactivation or decomposition was determined with different testing methods at temperatures of 71–120 °C and 199 °C for BHT and Vit E, respectively (Santos et al., 2012; Reda, 2011; Sanhueza et al., 2000). Moreover, degradation of Vit E in free form was already observed at low temperatures of 40 °C (Sabliov et al., 2009).

In addition, further effects of the antioxidants on the HME process were reported in the previously mentioned studies. Decreased glass transition temperature or reduced torque seems to be related to antioxidants that reduced degradation whereas increased torque was observed for antioxidants with ineffective stabilization properties (Crowley et al., 2002; Repka and McGinity, 2000). Consequently, a differentiation between the antioxidative effect and the effect of the modified melt properties by these additives is not feasible. Thus, related or improved results might be achievable by other processing aids such as plasticizers.

In this study, the stability of the drug was determined directly after the extrusion process. Further instabilities of dexamethasone that might occur during storage were not evaluated. In addition, HPLC analysis does not provide information on the crystalline structure of the undegraded drug, which can be affected by the thermal and mechanical forces during the HME process. The two known forms of the polymorphic drug dexamethasone differ only slightly in their melting behavior, but one is 1.5 times more water-soluble than the other (Oliveira et al., 2018; Varsa et al., 2022; Aljarah et al., 2019). Since this would have an impact on the drug release properties as well as on the bioavailability of the drug, such testing has to be involved in the further development process for the preferred formulation that meets the limits for degradation products.

In summary, the degradation of DEX is a risk factor when using thermal processing techniques such as HME or 3D printing. The application of processing temperatures as low as possible is the most effective method to avoid the degradation of the drug. This might be achievable by the addition of plasticizers. The use of supercritically CO_2_ as a temporary plasticizing agent would be a promising alternative due to its absence in the final product as already performed by various research groups (LaFountaine et al., 2016; Li et al., 2014). Furthermore, the residence time inside the extruder should be as low as possible to reduce the risk of drug degradation. In addition to screw speed adjustments, the conveying speed could be increased by varying the geometry of the screw elements (Douroumis, 2012).

## 5 Conclusions

This study on the stability of DEX during HME analyzed the effects of temperature, speed, polymer and additives for the manufacturing of EuRS, EC and PEO based filaments. The processing temperature was identified as the predominant parameter that forces the oxidative degradation of DEX. Fewer protective effects might be achieved by higher screw speeds due to shorter residence time of the drug in the extruder. The addition of the plasticizer PEG or TEC and the addition of the antioxidants BHT or Vit E did not result in considerable differences in DEX recovery in the tested conditions. However, differences were detected at the same processing temperatures for different tested polymers so the type of polymer used also seems to play a role in the degradation process.

HME of EuRS and EC was successful for plasticized and unplasticized compositions with DEX recovery above 96%^A^ at temperatures of 110–140 °C and 150–160 °C, respectively. The highest DEX recovery was about 92–96%^A^ for PEO based filaments at 140–150 °C. The DEX stability should be improved by reducing the processing temperature, potentially assisted by the addition of plasticizers when PEO is used as polymer base.

This study also demonstrated the importance of a suitable analytical method for the detection of degradation products necessary to ensure drug stability during thermal processing since the application of predictive methods for drug stability in these processes is limited. Furthermore, the results of this study and the findings reported in the literature highlight the complexity of HME-associated instabilities influenced by diverse parameters which cannot be clearly distinguished.

## Funding

This work was financially supported by the 10.13039/501100002347Federal Ministry of Education and Research of Germany (BMBF), “RESPONSE–Partnership for Innovation in Implant Technology” in the program “Zwanzig20– Partnership for Innovation”.

## CRediT authorship contribution statement

**Vanessa Domsta:** Writing – review & editing, Writing – original draft, Visualization, Supervision, Project administration, Methodology, Investigation, Formal analysis, Data curation, Conceptualization. **Tessa Boralewski:** Writing – review & editing, Investigation, Formal analysis, Data curation, Conceptualization. **Martin Ulbricht:** Writing – review & editing, Conceptualization. **Philipp Schick:** Writing – review & editing, Conceptualization. **Julius Krause:** Writing – review & editing, Conceptualization. **Anne Seidlitz:** Writing – review & editing, Supervision, Project administration, Funding acquisition, Conceptualization.

## Declaration of competing interest

The authors declare the following financial interests/personal relationships which may be considered as potential competing interests: Vanessa Domsta, Martin Ulbricht, Philipp Schick reports financial support was provided by Federal Ministry of Education and Research of Germany. Vanessa Domsta on behalf of all authors reports equipment, drugs, or supplies was provided by Evonik Industries AG, Essen, Germany. Vanessa Domsta on behalf of all authors reports equipment, drugs, or supplies was provided by DuPont, Wilmington, USA. Vanessa Domsta on behalf of all authors reports equipment, drugs, or supplies was provided by The Dow Chemical Company, Midland, USA. If there are other authors, they declare that they have no known competing financial interests or personal relationships that could have appeared to influence the work reported in this paper. Authors declared no conflict of interest. As previously stated, this work was financially supported by the 10.13039/501100002347Federal Ministry of Education and Research of Germany (BMBF), “RESPONSE–Partnership for Innovation in Implant Technology” in the program “Zwanzig20– Partnership for Innovation”. The authors thank Thomas Brand for his technical assistance and the suppliers of the used polymers for providing Eudragit®, ETHOCEL™ and POLYOX™ samples.

## Data Availability

Data will be made available on request.
